# Obituary

**Published:** 1919-12

**Authors:** 


### OBITUARY

Charles C. Voelker died in Trenton, N. J., September 13th of heart trouble. Dr. Voelker was well known for valuable work done by him on silicate cement. He was for a time an instructor in the University of Pennsylvania.

### Resolutions on the Death of Doctor Newell Sill Jenkins

*Whereas:* The members of the National Dental Association having learned of the death of Dr. Newell Sill Jenkins, and

*Whereas:* The name of Dr. Jenkins having been associated with all that was highest and best in dental practice for many years, and

*Whereas:* His long service in Europe and his wide knowledge of men and events having given him an international reputation second to none, and

*Whereas:* His delightful and charming personality added to his ability as a practitioner, made him a commanding figure in the profession;

*Therefore, be it Resolved:* That this Association places on record its high appreciation of his many sterling and lovable qualities, and its sorrow at his decease, and be it further

*Resolved:* That a copy of these resolutions, as an evidence of our affection and as an expression of our sympathy, be conveyed to the family of Dr. Jenkins, and preserved in the minutes of the Association.—Homer C. Brown, Chairman, Truman W. Brophy, Waldo E. Boardman, National Dental Association Committee.

